# Is safety in the eye of the beholder? Discrepancies between self-reported and proxied data on road safety behaviors—A systematic review

**DOI:** 10.3389/fpsyg.2022.964387

**Published:** 2022-09-02

**Authors:** Sergio A. Useche, Mireia Faus, Francisco Alonso

**Affiliations:** ^1^ESIC Business & Marketing School, Valencia, Spain; ^2^DATS (Development and Advising in Traffic Safety) Research Group, INTRAS (Research Institute on Traffic and Road Safety), University of Valencia, Valencia, Spain

**Keywords:** road safety, human factors, risk behavior, proxies, behavior, systematic review

## Abstract

Recent studies have problematized on the lack of agreement between self-reported and proxied data in the field of road safety-related behaviors. Overall, and although these studies are still scarce, most of them suggest that the way we perceive our own road behavior is systematically different from the perspective from which we perceive others' behavior, and vice versa. The aim of this review paper was to target the number and type of studies that have researched the behavioral perceptions of different groups of road users, contrasting self-reported behavioral data with those reported by other users (proxied), and their outcomes. This systematic review followed the PRISMA methodology, which allows for the identification of relevant articles based on the research term. A total number of 222 indexed articles were filtered, and a final selection of 19 articles directly addressing the issue was obtained. Search strategies were developed and conducted in MEDLINE, WOS, Scopus and APA databases. It is remarkable how road users perceive themselves as behaviorally “safer” than the rest of road users in what concerns the knowledge of traffic norms and their on-road performance. In addition, and regardless of the type of user used as a source, self-reported data suggest their perceived likelihood to suffer a traffic crash is lesser if compared to any other user. On the other hand, proxied reports tend to undervalue third users' performance, and to perceive riskier behaviors and crash-related risks among them. The outputs of this systematic review support the idea that the perception of road users' behavior and its related risks substantially differ according to the source. It is also necessary to increase the number, coverage and rigor of studies on this matter, perhaps through complementary and mixed measures, in order to properly understand and face the bias on road users' risk-related behaviors.

## Introduction

Typically, literature approaches the concept of “risk” as the probability of occurrence held by an event, or its potential consequences (or both of them), that are generally negative (Zio, 2018). In the majority of cases, human beings are somehow implied either in what causes the event or in suffering its consequences (Glendon et al., 2006). Further, risks are usually (an implicitly) appraised in terms of probability, referring in most cases to the likelihood of experiencing a negative outcome (Gao et al., 2015). Specifically in the field of traffic safety, the most common type of hazard typically perceived by road users are traffic crashes (AKA “accidents”).

It is known that ~1.35 million people die because of road traffic crashes every year (WHO, 2018). Road behaviors have gained ground during the last two decades as key contributors for traffic crashes, resulting in the development of behavioral-based approaches to assess road risks among different groups of users, such as: drivers—the most frequently addressed type of road users (Reason et al., 1990; Af Wåhlberg et al., 2015; Useche et al., 2017), motorcyclists and moped riders (Elliott et al., 2007; Steg and Van Brussel, 2009), bicycle riders (Feenstra et al., 2011; Hezaveh et al., 2018; Useche et al., 2018), and even pedestrians (Deb et al., 2017; Useche et al., 2020, 2021b).

Overall, the outcomes of these behavioral-based studies, most of them performed on the basis of behavioral self-reports, agree on the fact that risky behaviors may predict crash rates and/or causality-related consequences of crashes, such as crash severity and some potential ways to prevent crashes preceded by road misbehaviors. Another interesting highlight is that the level of risk perceived by some groups of users is rather inconstant, and can largely vary depending on their personal and social characteristics (Hansson, 2010; Ngueutsa and Kouabenan, 2017).

### Heterogeneity and bias in safety-related behavioral perception

Literature sources largely agree on the fact that perception is a considerably subjective and multidimensional issue that cannot be separated from the social-environmental context where road behaviors take place (Keage and Loetscher, 2018). Therefore, there might be many factors directly influencing perception, e.g., previous experiences (Lujala et al., 2015), values, norms, cultural contexts (Venuleo et al., 2017), awareness and credibility of hazards (Van Zoonen and Van der Meer, 2015), uncertainty (Karl, 2018), emotional conditions (Hu et al., 2015), and cognitive and heuristic biases (Tsohou et al., 2015). Some of which can be considered by researchers during data collection and analysis phases, but can hardly all be controlled.

Although most empirical studies in the matter support the idea that road user behave in a way that is consistent and coherent with their personal perception (Sheeran et al., 2014; Nasaescu et al., 2020), it is difficult to prove the strength of the consistence between predisposition factors and actual behaviors (Af Wåhlberg et al., 2015). In this regard, it is safe to say that having a greater risk perception (adjusted to the real dangerousness of the event) is essential to “guarantee,” to some extent, a reduced likelihood to perform risky behaviors (Ferrer and Klein, 2015). However, if cognitive bias are introduced, behavioral adjustments will hardly happen: people tend to either overestimate or underestimate the probability of an issue happening, even though their own behavior and integrity might be compromised if, for instance, a risk is assumed (Lodder et al., 2016). This is, perhaps, one of the main shortcomings present in the behavioral-based research of traffic crashes; road users with both positive and negative attitudes, perceptions or predispositions toward road safety may actually engage in risky behaviors such as exceeding speed limits, drinking alcohol, or talking on the phone while driving, although to a different extent (Ram and Chand, 2016).

Specifically in regard to choosing “the most reliable” data sources on the field of road safety behaviors, it is worth arguing that source suitability might largely depend on the person whose behavior is reported. In other words, the perspective from which road users perceive their own behaviors becomes systematically different from the way they perceive other users' ones. Cullen et al. (2015), have shown how individuals tend to have different information on ourselves than we have on others, and this may lead to different interpretations of the perceived events.

Also, empirical literature has endorsed the assumption that a perceived action will have a stronger affective feeling if it is performed by oneself rather than by someone else (Hommel, 2018). This tends to strengthen cognitive biases, such as the *Lake Wobegon effect*, which basically consists of overestimating one's own positive abilities, and underestimating negative qualities instead (Lim, 2019). This contributes to establishing a difference in the perception of one's own behaviors and the ones performed by other people, generally holding a more favorable opinion of oneself, which can be non-adjusted to reality (Vickers and Kent, 2015).

Seen differently, this discrepancy proves to be relevant in the traffic and road safety field, where overestimating one's own behaviors can have severe consequences such as traffic crashes (Cuenen et al., 2015). Mostly, studies in the field have focused on assessing the self-reported behaviors of users. However, there is a scarcity of literature contrasting the perception of users about themselves and about potential hazards with their perception of these elements in other road users.

In view of the aforementioned considerations, it is worth mentioning that the number of studies addressing the accordance/discordance between road users' behavioral data sources is yet undetermined.

Therefore, the aim of the present systematic review was to identify the number and type of studies that have researched the behaviors and attitudes of road users, in which the self-reported perceptions were contrasted with other users' perception on a specific road group. The core hypothesis of this review was that there would be a discrepancy in the perception of road safety-related behaviors depending on the group of road users. In addition, we estimated that the self-reported behavior would be better perceived by other road users, “better” meaning “more adequate,” with fewer infractions and fewer risky behaviors.

## Methods

### Study setting

Systematic reviews have been described as a process of mapping the existing literature, using a transparent and systematic process to define a research question, search for studies, assess their quality and synthesize findings, either qualitatively or quantitatively (Armstrong et al., 2011).

To conduct this systematic review, and given its parsimonious structure, we used the Arksey and O'Malley (2005) methodology. This framework provides recommendations and successive steps useful to clarify and enhance each stage of the review process. The five stages suggested by Arksey and O'Malley (2005) are:

(1) Identifying the Research Question,
(2) Finding Relevant Studies,
(3) Selecting the Studies,
(4) Charting the Data and Collating,
(5) Summarizing, and Reporting the Results.

#### Step 1: Identifying the research question

As previously mentioned, the purpose of the present scope review was to identify the number and type of studies researching risk perception, behaviors, and attitudes of different road users. In addition, their self-reported narratives with those manifested in other users was contrasted.

Since specialized literature claims that risky road (human) behavior is the primary predictor of crashes (Stephens et al., 2017; Puchades et al., 2018), road users' perspectives must be investigated. In this sense, we seek to find common patterns or trends that explain the discrepancies (or concordances) in the assessment of the different groups of road users.

After a first research—and considering the scarcity of studies available–, we opted for selecting all research focused on this topic, regardless of whether users were the objective of the study or not. Therefore, the population consisted of drivers, cyclists, pedestrians, and any other type of road users. The results included a summary and a topic analyses of all the chosen articles.

#### Step 2: Finding relevant studies

The present review was carried out following the PRISMA guidelines for the notification of systematic reviews (Moher et al., 2011). PRISMA begins the process by looking for records in each of the databases that were found during the searches. It then moves on to the overall number of records after removing duplicates, and finally to the individual studies in the qualitative and quantitative synthesis (Urrútia and Bonfill, 2010). This technique, which allows for an organized (but flexible) set of stages to be followed, has been widely employed in numerous research and systematic reviews on a variety of topics, including human behavior and traffic crashes in the context of various groups of road users (Heidari et al., 2019; Oviedo-Trespalacios et al., 2019).

The databases that were used for the preliminary search of the literature were the Web of Science, American Psychological Association (APA), Scopus and MEDLINE. These databases were chosen for their vast quantity of publications and their connection to behavioral-based studies, particularly in the domains of psychology, behavioral sciences, and practical road safety (Azami-Aghdash et al., 2018; Malakoutikhah et al., 2021). Other lists of systematic and extensive reviews of other primary research papers, which were theoretically eligible but not collected by our search engines, were also evaluated to identify possibly appropriate studies not indexed within the aforementioned data sources.

The search included literature published from the beginning of the database, and included the third week of February 2022. The terms we searched for included: “discrepancy of perception,” “oneself and others,” “drivers and non-drivers,” “drivers and pedestrians,” “cyclists,” “behavior,” “attitudes,” and “road users.” These terms were identified after a review of the titles and keywords of the articles we found during our preliminary search.

#### Step 3: Selecting the studies

Articles were excluded during this stage if they did not refer to our research objective by contrasting the perception of behavior and/or attitudes of different road users. Publications in the form of conferences/summaries, protocols, letters, editorials, case reports or case series were not selected. We also restricted our eligibility criteria to articles published in English and Spanish, publicly available or possibly requestable from the library system that was being used.

Initially, all authors independently assessed a subset of titles and summaries, and then met up in order to discuss and solve any discrepancies. This is a common approach in systematic reviews addressing road behavioral-based research. It covers and/or compares the case of various groups of users, which has been done in recent years with reviews on road user behaviors (Moran et al., 2019).

#### Step 4: Charting the data

The articles that fitted the inclusion criteria were critically reviewed using the descriptive-analytic Arksey and O'Malley (2005) method. For each eligible article that was included, the following data were extracted and registered: title of the article, author(s), year of publication, country of the study, study design, group of users that was analyzed, sample size, main findings and results (discrepancy/agreement found between groups). Similar information from the selected papers has been reported in prior systematic reviews regarding road user behavior (Spindler et al., 2018; Schönbach et al., 2020).

#### Step 5: Collating, summarizing, and reporting the results

The graphed data were summarized in tables and followed by the descriptive data, analyzed through a thematic-based organization strategy. For this purpose, papers were analyzed in the light of the column-based structure presented in Table 1.

**Table 1:** General characteristics of eligible studies.

**References**	**Country**	**Study aim(s) and setting**	**Users covered**	**Method**	**Results (main outcomes)**	**Key limitations**
James et al. (2019)	United States	The study use an online survey to evaluate the perceived safety and sidewalk blocking of e-scooters.	Pedestrian and cyclists (*n =* 181)	Cross-sectional	Pedestrians perceived greater safety around bicycles than e-scooters. Different road users are not accustomed to the presence of e-scooters as a means of transportation in cities.	(1) Self-report (2) Local coverage (3) Small sample
Castanier et al. (2012)	France	The study used a questionnaire to assess risk perceptions related to interactions between different road users and streetcars. Specifically, self-reported behavior and knowledge of regulations.	Pedestrians (*n =* 379), cyclists (*n =* 146), and car drivers (448)	Cross-sectional	All three types of road users perceive a very low risk of collision between a streetcar and themselves. And they consider that the collision is more likely to be with other road users. There was realistic optimism among pedestrians and unrealistic optimism among drivers.	(1) Self-report (4) Not representative (5) Not generalizable
Kaparias et al. (2012)	United Kingdom	The research analyzes the importance of some person-, context- and design-specific factors that modulate drivers' and pedestrians' perceptions of shared road space	Pedestrians and car drivers (*n =* 871)	Cross-sectional	Pedestrians feel safer when they are perceived as visible to other road users (specifically in conditions of high pedestrian traffic, low vehicle traffic, good road lighting and pedestrian-only facilities). Drivers are more uncomfortable when the road is occupied by many pedestrians (especially if they are children and the elderly).	(1) Self-report
Useche et al. (2021a)	Spain	The study analyzes the differences between cyclists' self-reported behavior and other road users' (non-cyclists) perceived behavior of cyclists through the ECBQ questionnaire.	Cyclists (*n =* 1,064) and non-cyclists (*n =* 1,070)	Cross-sectional	Non-cyclist users state that cyclists engage in riskier behaviors than they self-report. Thus, the self-reported and proxy-reported behaviors of cyclists differ greatly in terms of traffic violations, driving errors, and positive behaviors.	(1) Self-report (5) Not generalizable
Chaurand and Delhomme (2013)	France	The research assesses the perception of risk in interactions between cyclists and motor vehicles, in addition to measuring the perceived risk of collision in road situations where cyclist collisions are frequent.	Cyclists (*n =* 336) and car drivers (*n =* 92)	Cross-sectional	Drivers perceive more risk than cyclists in their interactions. A perception influenced by the user experience variable and by the degree of control of the situation in both drivers and cyclists.	(1) Self-report (3) Small sample (4) Not representative (6) Data limitations
Arai et al. (2010)	Japan	The research aimed to analyze perceptions of driving and to examine differences in perceptions according to age and driving status.	Car drivers and non-car drivers (*n =* 1,010)	Cross-sectional	Drivers state that “driving is a “right” that we all deserve”, while non-drivers do not share this statement. Personal mobility is the main reason for drivers not to stop using motor vehicles.	(1) Self-report (5) Not generalizable
García-Ramírez (2018)	Ecuador	The study evaluates the perception of road users through a survey on sensations, emotions and behaviors as road users, as well as their opinion regarding crashes and current traffic laws.	Pedestrians and drivers (*n =* 1,197)	Cross-sectional	Most drivers feel annoyed by the way others drive, but justify their own risky maneuvers. Most pedestrians feel fear when driving on the street, while passengers or co-drivers feel fear and stress. Traffic laws are not supported by a large proportion of respondents.	(1) Self-report
Horswill and McKenna (2006)	United Kingdom	An experiment was conducted to find out the effect of perceived control on risk-taking in a dynamic, everyday task.	Drivers and passengers (*n =* 96)	Cross-sectional and Experimental	Drivers chose faster speeds and took more risks than passengers.	(3) Small sample
Wood et al. (2009)	Australia	A survey was administered on the attitudes and behaviors of road users in relation to visibility problems.	Drivers (*n =* 662) and cyclists (*n =* 838)	Cross-sectional	Cyclists believe that they are visible at greater distances than drivers thought, and they believe they are more visible when using bicycle lights than drivers report. There are discrepancies between the groups regarding the most effective visibility measures.	(1) Self-report
Almannaa et al. (2021)	Saudi Arabia	The study investigates the feasibility of launching an e-scooter sharing system as a new mode of micro-mobility, and part of the public transport system with respect to the mobility and perception of the e-scooter.	Cyclists and non-cyclists (*n =* 439)	Cross-sectional	Eighty-two percent of people who have previously used e-scooters reported that they consider e-scooters to be a safe or potentially safe mode of transportation. However, 90% of respondents who believe e-scooters are unsafe have never used one.	(1) Self-report (4) Not representative (5) Not generalizable
Paschalidis et al. (2016)	Greece	The responsibility for crashes in urban space attributed by the cycling population was investigated.	Cyclists, pedestrians and car drivers (*n =* 306)	Cross-sectional	Cyclists who are also drivers tend to blame pedestrians in crashes, especially in incidents that occur on shared-use paths.	(1) Self-report (5) Not generalizable (6) Data limitations
Sullman and Taylor (2010)	United Kingdom	A questionnaire on driving accidents and incidents in the last year was administered, with the self-report scales DRAS, DBQ and BIDR.	Drivers (*n =* 1,307)	Longitudinal	Social desirability had no or little effect on the DRAS. However, some items assessing general avoidance were higher in the public setting, which may be linked to the effect of socially desirable responding on driving avoidance due to environmental or practical concern.	(1) Self-report (4) Not representative
Thomas and Walton (2007)	New Zeeland	The study analyzes observed hand positions on the steering wheel and hand placements on the steering wheel reported by a sample of SUV and car drivers.	Drivers (*n =* 1,196)	Cross-sectional	Observed hand positions reveal that SUV drivers are more likely to drive with one hand instead of two hands on the top half of the steering wheel, indicating a lower level of perceived risk.	(6) Data limitations
Corbett (2001)	United Kingdom	The study consisted of several field experiments in which deployment strategies were introduced and samples of drivers were surveyed once or twice before and/or after the experimental manipulations.	Drivers	Cross-sectional/Longitudinal and Experimental	The results supports others that have confirmed the existence of a significant relationship between self-reported and observed measures of speeding, but has revealed a tendency for faster drivers to underestimate their normal speed and slower drivers to exaggerate theirs.	(1) Self-report
Blanchard et al. (2010)	Canada	Electronic tracking devices (CarChip and GPS) were used. Drivers completed trip logs, diaries, a questionnaire on habitual driving habits, ratings of frequency and avoidance of driving situations, and a follow-up interview.	Drivers (*n =* 61)	Cross-sectional/Longitudinal	Older drivers' self-estimates of distance traveled are inaccurate.	(3) Small sample (4) Not representative (5) Not generalizable
Reimer et al. (2006)	United Kingdom	The study compared observed driving simulator behaviors and self-reported measures of driving behaviors to ascertain the degree of agreement.	Drivers	Cross-sectional and Experimental	The results indicated that the data collected are valid measurements, although they were not exactly identical.	(1) Self-report
Van Huysduynen et al. (2018)	The Netherlands	Study participants were required to complete the MDSI questionnaire and drive in the driving simulator.	Drivers (*n =* 88)	Cross-sectional and Experimental	Self-reported driving style correlates with actual driving behavior in a driving simulator for careful, risky, and furious driving. However, no evidence is manifested that anxious, dissociative, and distress-reducing self-reported driving styles correlate with driving behavior.	(3) Small sample (4) Not representative (5) Not generalizable
Johnson et al. (2014)	Australia	The research assessed behaviors, knowledge and attitudes through an online survey.	Drivers and cyclists (*n =* 1,984)	Cross-sectional	Cyclists were more likely to self-report safer driving behavior and better attitudes toward cyclists compared to non-cycling drivers.	(1) Self-report
Rowden et al. (2016)	Australia	The research measured risk behaviors through a survey of different types of road users.	Drivers and motorcycle riders (*n =* 438)	Cross-sectional	Self-reported aggressive behaviors were higher in drivers than in motorcyclists, manifesting feelings of anger and frustration.	(1) Self-report

## Results

### Search results

**Figure 1:**
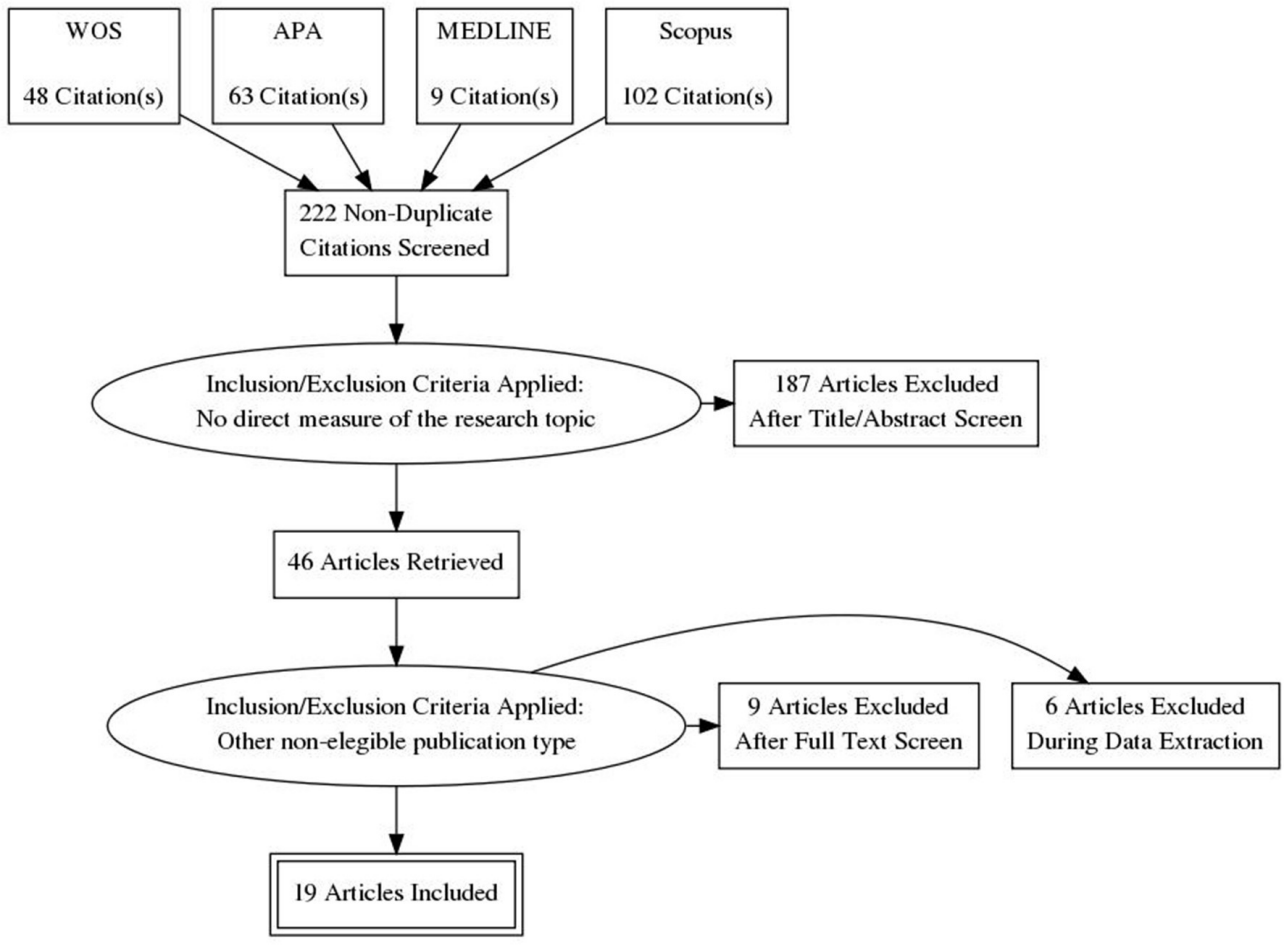
PRISMA diagram. WOS, Web of Science; APA, American Psychological Association.

Once the doubled (duplicate papers) or non-accessible elements were ruled out, the searched words identified a total number of 222 possible articles. A manual selection of the articles that adjusted to the objective of the review left us with 19 eligible articles. Figure 1 shows the data source searching and selection process.

### Characteristics of eligible research articles

**Figure 2:**
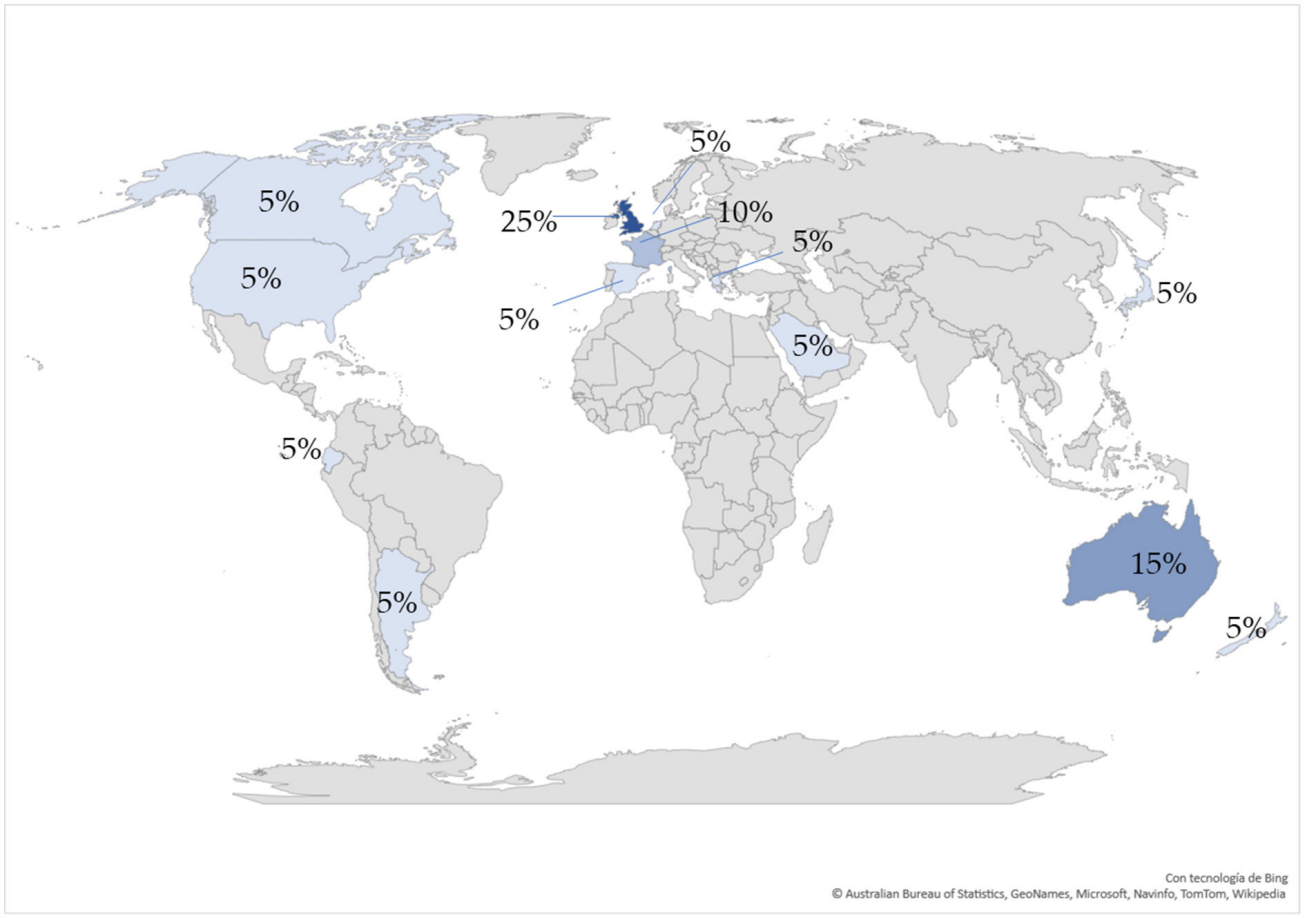
Geographical distribution (country of origin) of the selected studies.

Our search did not have time limits, therefore, there were 19 studies complying with the inclusion criteria, published in English between 2001 and 2022, which indicates that the study topic is a recent one. Moreover, the studies were conducted in different countries. Thus, there are 13 countries represented: United Kingdom (*n* = 5), Australia (*n* = 3), France (*n* = 2), Spain (*n* = 1), Greece (*n* = 1), Japan (*n* = 1), Saudi Arabia (*n* = 1), United States (*n* = 1), Canada (*n* = 1), The Netherlands (*n* = 1), New Zeeland (*n* = 1), and Ecuador (*n* = 1) (Figure 2).

A huge variability can be found in the types of users studied in this research. There is only one case in which the perceptions of pedestrians, cyclists and drivers were contrasted. The most repeated comparisons were between pedestrians and drivers (*n* = 3), cyclists and non-cyclists (*n* = 2), and cyclists and drivers (*n* = 3). In the rest of cases, when road groups were contrasted, there was only one study contrasting pedestrians and cyclists, drivers and non-drivers, drivers and vehicle passengers, drivers and motorcycle riders, and cyclists compared to pedestrians and drivers.

Considering the scarcity of publications contrasting the same group of users, research that contrasted the self-perception of users with objective data was included, thus assessing their level of adjustment to real behaviors. There are 6 studies where the self-reported perception of drivers was compared, either through objective measurements of their behavior and attitudes assessed by means of their habitual trajectories, or through the use of simulators.

However, and closely related to idiosyncratic issues commonly affecting this type of studies, the design was cross-sectional in most of them where the researchers measured the variables without intervening. Thus, in these cases the use of a questionnaire was unanimous. It was what measured the assessments or perceptions of users regarding themselves or other road groups. The experimental studies were applied, in the majority of cases, only when drivers were analyzed. However, only one case was found of experimental design where road groups played a role. In this study, the analyzed element was the perception of drivers and passengers, since the sample had been divided in two groups that were showed a different video each before undergoing the tests.

For what concerns the approached topics, we can perceive a lot of variability. Nevertheless, they can be framed in three groups: perception of safety and road interactions, perception of the knowledge and compliance with norms, and perception of risky behaviors and traffic crashes.

## Discussion

The core aim of this study was to assess the number and type of studies that have researched behavioral perceptions of different groups of road users. This was achieved contrasting self-reported behavioral data with those reported by other users (proxied), and their conclusions in these regards.

A first highlight provided by this systematic review is the scarcity of empirical research delving into individual perceptions on other road users' road behavior, i.e., proxied behavioral assessments. Rather, and as often observed in the field of traffic psychology, key issues such as road safety (and risky) behavior (e.g., Devlin and McGillivray, 2016; Meader et al., 2016), personality factors or traits (Guo et al., 2016; Zicat et al., 2018) and risk perception (Hoffmann et al., 2015; Oviedo-Trespalacios et al., 2017) have been predominantly assessed through self-reported data collection methods. This means that in current empirical literature on the matter, study participants commonly provide assessments on their own behaviors. However, it is really exceptional the case which the accordance/discrepancy between proxied and self-reported road behaviors is assessed.

In this regard, some studies have problematized on both the extent and high degree of the existing discrepancies between behavioral self-reports and the actually observed road risky behaviors of individuals. Thus, shedding light on the overestimation of one's own abilities as a predominant pattern (Af Wåhlberg et al., 2015; Chen et al., 2020). In fact, in this systematic review, some of the studies included clearly show the existing differences between self-reported behaviors and real behaviors, thus manifesting biases in the self-perception of drivers (Sullman and Taylor, 2010). Also, this pattern shows a certain level of concordance with the one found by studies that compare perceptions from different road users regarding risky or safety behaviors of single types of road users. In this type of studies, self-reported road behaviors are largely positive when road users grade their own behavior, while external raters' assessment tends to be comparatively critical (Wood et al., 2009; Arai et al., 2010; Li et al., 2016; Duarte and Mouro, 2019; Useche et al., 2021a).

### Filling the source-related gap: The need of crossing data

In this sense, further development of research addressing other users' point of view of specific groups of road users could be interesting. This would enhance the measurement of the level of accordance/discordance between human data sources on road safety issues. A first light given by the literature is provided by the Weiner's *attribution theory* (Heider, 1958; Weiner, 2010), which states that people need to know the cause of the behaviors they observe or perform, since they use “common sense inference rules,” that might operate differently depending on who is being judged.

Moreover, other studies have illustrated how behaviors tend to be consistently attributed to certain (external or internal) causes. Therefore, fixed ideas and other acquired predispositions become potential sources of biased assessments and predictions (Eberly et al., 2011). For example, a person who assumes that “*young cyclists behave irresponsibly*” has a good chance of predicting that cyclists' reckless behavior will remain over time, even if it is considered that in 5–10 years young cyclists will not be the same. Therefore, personal interpretations and descriptions of the actions of other road users could be consequently linked to cognitive biases based in subjective experiences (Concha et al., 2012), being this a matter that traffic psychology should further develop.

Focusing on the available studies, it can be observed that users tend to have a better perception of themselves than of the rest of people. The study that compares the perceptions of drivers and passengers (Horswill and McKenna, 2006) is especially clarifying in this aspect. In this case, researchers gave each participant one role during the experiment. The results suggest that passengers considered the hypothetical driver less capable of dealing with high speed than themselves. Therefore, this fits into the evidence related to the optimism bias, in which drivers tend to believe that they are better than average (González-Iglesias et al., 2015; Nees, 2019). Thus, it can be deduced that, their perceptions on other road users may vary depending on the role they assume while considering themselves the ones most in control of the situation.

### Risk-related perceptions on crash likelihood

This study also allowed us to notice that—in current literature—, there is often reported the fact that individuals believe they have fewer probabilities of suffering a traffic crash than others. In this sense, this phenomenon is also determined by the belief that one has more experience and/or capacities than the rest of a certain group of road users (Meng et al., 2015). Thus, one will be prone to avoid risky situations, and in case of finding him/herself in one, he/she will be able to escape without suffering a crash, which is quite unrealistic in many cases (Stavrinos et al., 2016).

Given the subjective nature of risk perception in traffic, it is often assessed in function of the available information, which makes it highly individual and dependent on previous crash-related experiences (Machado-León et al., 2016; Eboli et al., 2017). In this sense, interventions and campaigns that are adjusted to the perceived risk, and to the real dangers of a situation need to be developed (Faus et al., 2021). Paradoxically, an excess of self-confidence and downplaying suffering a crash are, indeed, risk factors for being involved in one (Cordellieri et al., 2016). Also, and coherent with the reviewed literature, the results of this review endorse the assumption that the overestimation of one's own abilities can lead to perform certain risk assumptions and risky behaviors, thus leading to road hazards (Devlin and McGillivray, 2016). One of the key factors for learning hazard prevention mechanisms is to be aware that causalities can occur (Ferrer and Klein, 2015). Therefore, it might be necessary to influence and highlight the importance of the human factor in crashes.

Coherently, and although much more action remains pending in this regard, some studies have added certain highlights on the fact that giving feedback on the performance of drivers is positive for users, who then manifest better attitudes and behaviors during subsequent trips (Horswill et al., 2017). On one hand, influencing protection and prevention elements (and habits) can directly affect the likelihood of being involved in a crash (or at least in the behaviors preceding them; Useche et al., 2019).

On the other hand, methodological shortcomings on behavioral road-risk assessments seem to remain a key issue to consider, being a first step to increase the reliability and validity of studies on this matter.

### Limitations and further research

This scoping review was carried out considering a large set of potential sources retrieved through relevant indexes and databases worldwide. However, the final number of original research papers accomplishing the selection criteria was considerably reduced. On one hand, it is true that this is (indeed) one of the conclusions to provide, i.e., that the literature on this matter is really scarce. On the other hand, it could considerably limit the broadness and scope of the other conclusions presented. Also, it is possible that non-indexed literature (regardless on the different discussions on the validity of the findings it could append) may provide further data on this important and understudied research problem.

As for further studies, research contrasting behavioral-related perceptions from different groups of road users could provide important highlights for understanding the actual behavior of users. Concretely, most of the existing research exclusively focuses on retrieving data on user's risky (but not on positive) behaviors. Therefore, protective behaviors could be addressed in future research experiences on this matter. Future studies could also try to integrate mixed data sources and methods more holistically while addressing behavioral contributors to traffic crashes among different groups of road users.

## Conclusion

The results of this systematic review, apart from remarking the problematic scarcity of literature in this regard, suggest that the extent to which road users' behavior is perceived as safe highly depends on the individual assessed.

Furthermore, road behaviors from third parties are commonly perceived as “riskier,” while own-behavioral assessments tend to be more positive, undervaluing own road-risk assumptions and misbehaviors. Also, proxied reports tend to undervalue third users' performance, thus assuming greater crash-related risks among them.

The findings of this systematic review can have theoretical and practical implications for multiple entities and sectors of activity:

- Government authorities and public and private entities related to traffic, mobility and road safety can use the information provided to understand road users' behaviors and beliefs, and for the development of more effective preventive measures, which take into account road users' cognitive biases.
- Companies and organizations responsible for road safety education programmes, communication campaigns and social advertising can also benefit from the results of the study. This information could be used to develop actions that emphasize the possible distortions of drivers and other road users, and the impact that an unrealistic risk perception can have on the performance of risky behavior on the road and, consequently, on road accident rates.

In conclusion, it seems necessary to increase the number, coverage and rigor of studies on this matter. This could be achieved through complementary and mixed measures, in order to properly understand and face the bias on road users' risk-related behaviors, and thus that might contribute to reduce them.

## Data availability statement

The original contributions presented in the study are included in the article/supplementary material, further inquiries can be directed to the corresponding author/s.

## Author contributions

SU: conceptualization, methodology, data analysis, investigation, writing—original draft preparation, and writing—reviewing and editing. MF: writing—original draft preparation and writing—reviewing and editing. FA: visualization and supervision. All authors contributed to the article and approved the submitted version.

## Funding

This work was supported by the research grant ACIF/2020/035 (MF) from Generalitat Valenciana. Funding entities did not contribute to the study design or data collection, analysis and interpretation, or writing of the manuscript.

## Conflict of interest

The authors declare that the research was conducted in the absence of any commercial or financial relationships that could be construed as a potential conflict of interest.

## Publisher's note

All claims expressed in this article are solely those of the authors and do not necessarily represent those of their affiliated organizations, or those of the publisher, the editors and the reviewers. Any product that may be evaluated in this article, or claim that may be made by its manufacturer, is not guaranteed or endorsed by the publisher.
